# The triglyceride-glucose index and contrast-induced nephropathy in non-ST elevation myocardial infarction patients undergoing percutaneous coronary intervention

**DOI:** 10.1097/MD.0000000000032629

**Published:** 2023-01-13

**Authors:** Erol Gursoy, Onur Baydar

**Affiliations:** a Koc University Hospital, Department of Cardiology, Istanbul, Turkey.

**Keywords:** myocardial infarction, nephropathy, the triglyceride-glucose index

## Abstract

The triglyceride glucose (TyG) index is an indicator of insulin resistance and associated with increased risk of diabetes mellitus and cardiovascular events. Our study investigates the correlation between TyG index and contrast induced nephropathy (CIN) in non-diabetic patients with non-ST elevation myocardial infarction (NSTEMI) undergoing percutaneous coronary intervention (PCI). 350 non-diabetic NSTEMI patients (183, 52.3% male) undergoing PCI were retrospectively enrolled. The enrolled cohort was divided into 2 groups based on the calculated TyG index, namely values < 8.65 or higher. CIN was defined as an increase in serum creatinine > 25% or 0.5 mg/dL from baseline in the first 48 to 72 hours after PCI. A total of 56 (16%) cases of CIN were diagnosed. In contrast to patients with lower TyG indexes, patients with higher TyG indexes (≥8.65) had a higher frequency of CIN, 9.5%. versus 20.8% respectively (*P* .004). Patients with CIN also had higher TyG indexes (8.74 ± 0.12 vs 8.67 ± 0.11, *P* < .001). In addition, TyG index, age, and glomerular filtration rate were identified as independent risk factors for CIN in logistic regression model (OR: 2.5 CI: 1.3–4.6, *P* .006, OR: 1.0 CI: 1.0–1.1, *P* < .001, OR: 1.0 KI: 1.03–1.06, *P* .025). In the ROC analysis, the area under the curve predictive of CIN was 0.666 (*P* < .001, 95% [CI] 0.58–0.75) with a cutoff value of 8.69 (sensitivity 71,4%, specificity 55.1%) TyG index. Higher TyG indexes are associated with an increased risk of CIN in non-diabetic patients with NSTEMI.

## 1. Introduction

Percutaneous coronary intervention (PCI) decreases ischemic complications and increases survival in non-ST elevation myocardial infarction (NSTEMI) patients. contrast-induced nephropathy (CIN) refers to an acute renal complication after intravenous contrast agent administration during angiography. Patients undergoing PCI are more likely to develop CIN, which is linked to renal failure, extended hospital admissions, an increase in cardiovascular events, and death.^[1,2]^ Numerous factors may contribute to the development of CIN, including hypovolemia, amount of contrast, glomerular filtration rate (GFR) at onset, medications, and comorbidities.^[3,4]^ A recent study reported the incidence of CIN to be 19.7% in 31,532 acute myocardial infarction patients.^[5,6]^ Therefore, it is crucial to identify NSTEMI patients undergoing PCI who are at risk for CIN. Insulin resistance (IR) is a significant pathophysiological process and risk factor for diabetes mellitus (DM).^[7,8]^ IR is closely associated with the prevalence of chronic renal impairment.^[9]^ A recent study examining the incidence of CIN in patients with IR, as defined by the homeostasis model assessment of insulin resistance (HOMA-IR), showed IR to be an independent risk factor for CIN.^[10]^ The triglyceride glucose (TyG) index has been shown to be closely related to insulin resistance.^[11]^ As a new index to assess insulin sensitivity, TyG is strongly correlated with the HOMA-IR assessment model.^[12,13]^ The TyG index has been shown to be associated with many cardiovascular diseases.^[14–17]^ The TyG index has emerged as a promising alternative index of insulin resistance due to its efficacy and low cost. The aim of this study was to investigate the predictive value of the TyG index for the development of CIN in non-diabetic patients with NSTEMI undergoing coronary angiography.

## 2. Methods

We retrospectively followed 350 consecutive NSTEMI patients who underwent PCI between January 2014 and January 2018 in the cardiac department of Avicenna Hospital. Non-ST elevation myocardial infarction was defined according to current guidelines.^[13]^ All patients received aspirin (300 mg), a P2Y12 antagonist (clopidogrel 600 mg, prasugrel 60 mg, or ticagrelor 180 mg), and dual antiplatelet therapy (aspirin 100 mg daily plus clopidogrel 75 mg daily or prasugrel 10 mg daily or ticagrelor 180 mg daily) for at least 12 months. All PCI procedures were performed using unfractionated heparin (70–100 U/kg). Transthoracic echocardiography (Vivid 3; GE Medical System, I, Norway) was performed on all patients, and the Simpson method was used to calculate left ventricular ejection fraction. Patients with severe valvular heart disease, severe or congestive heart failure, known DM, treatment with anti-diabetics, intra-aortic balloon pressure support, severe renal failure, and emergency cardiac revascularization were excluded. This study was approved by the local ethics committee. Fasting lipid profiles, blood glucose, plasma creatinine, total cholesterol, high-density lipoprotein C, and triglyceride levels were measured enzymatically (Roche Diagnostics GmbH, Mannheim, Germany), and low-density lipoprotein C levels were calculated from lipid parameters. The TyG index was calculated using the formula log fasting TG × fasting glucose/2.^[18]^ Patients were he divided into 2 groups according to the TyG index. The first group included patients with her TyG index < 8.65 and a second group included patients with a mean TyG index > or equal to her 8.65. The cutoff value is based on the International Diabetes Federation definition.^[19,20]^

Hypertension was defined as blood pressure > 140/90 mm Hg, or treatment with antihypertensive drugs. In addition, DM was defined as fasting blood glucose > 126 mg/dL or being treated with oral antidiabetic drugs or insulin. Serum creatinine concentrations were monitored on admission, daily for several days thereafter, and at discharge. GFR was calculated using the modified formula of Levey et al is calculated.^[16]^ CIN was defined as a 25% or 0.5 mg/dL increase in creatinine from baseline within 48 to 72 hours after PCI.^[21]^

### 2.1. Statistical analysis

All analyzes were performed using SPSS Version 22 for Windows (SPSS Inc, Chicago, IL). Numeric variables are presented as mean standard deviation and nominal values are presented as percentages. The Kolmogorov-Smirnov test was used to determine whether each variable was normally distributed. The values of continuous variables were compared between the 2 groups by using independent-samples *t* tests. Non-parametric values were compared using the Mann–Whitney U test. Categorical data were compared using the chi-square test. To assess the impact of several factors on CIN development, we performed multivariate regression analysis using the backward logistic regression (LR) method. A variable with an unadjusted *P* < .05 was considered significant. Receiver operating characteristic curve analysis was used to determine the optimal cutoff value for the presence of TyG index and the number of TyG indexes to predict the development of CIN.

## 3. Results

**Figure 1.**
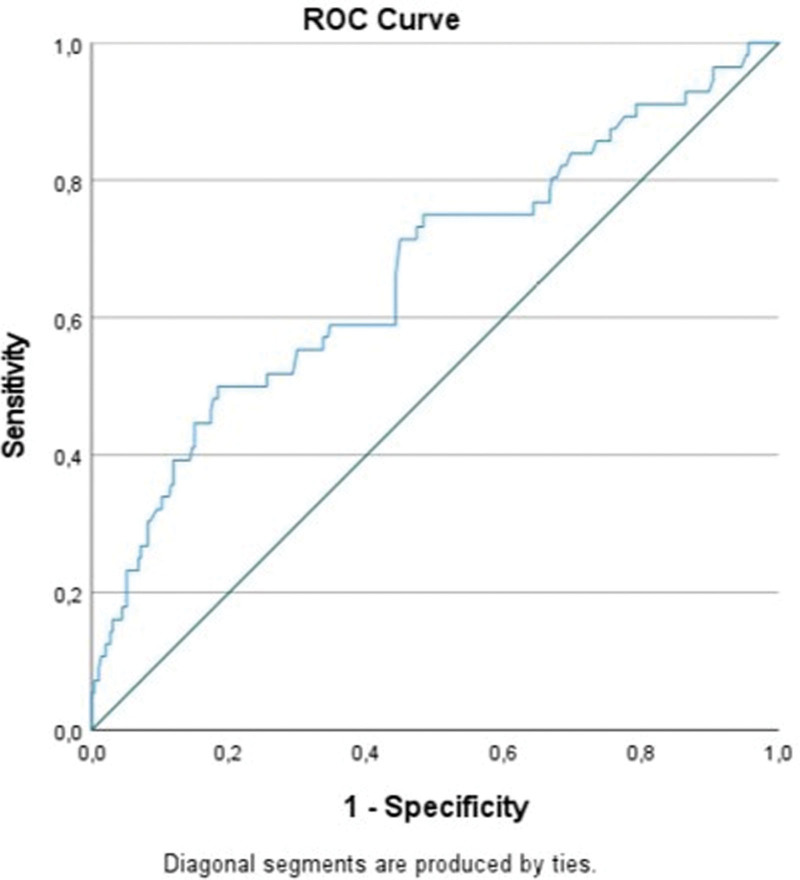
ROC curve analysis for TyG index. TyG = triglyceride glucose.

350 non-diabetic NSTEMI patients (183 men, 52.3% men) undergoing PCI were retrospectively enrolled. There were no significant differences between the 2 study groups with respect to weight, age, previous cerebrovascular disease, hypertension and HL. Grace score, peak troponin levels, serum glucose and low-density lipoprotein levels, time to reperfusion, contrast dose, left ventricular ejection fraction and eGFR did not differ between the 2 groups. However, high-density lipoprotein levels were significantly lower and TG levels were significantly higher in the high-score group. In addition, the 2 groups did not show difference in terms of in-hospital mortality, length of stay, major bleeding, need for ventilator, and dialysis. Patient baseline clinical and procedural characteristics are shown in Tables 1, 2, and 3. A total of 56 (16%) cases of her CIN were diagnosed. Comparing patients with a TyG index < 8.65 with those with a TyG index ≥ 8.65, higher-scoring patients had a increased frequency of CIN (42) 20.8% versus (14) 9.5%. *P* .004. Patients were then assigned to 2 groups according to the presence or absence of CIN. Patients with CIN also had higher TyG index values (8.74 ± 0.12 vs 8.67 ± 0.11, *P* < .001). In addition, TyG index, age, and eGFR were identified as independent risk factors for CIN in logistic regression analysis (OR: 2.5 CI: 1.3–4.6, *P* .006, OR: 1.0 CI: 1.0–1.1, *P* < .001, OR: 1.0 KI: 1.03–1.06, *P* .025). Analysis of characteristic receiver operating curves, area under the curve predicting CIN 0.666 (*P* < .001, 95% confidence interval [CI] 0.58–0.75), cutoff value 8.69 (sensitivity 71.4%, specificity 55.1%) the number of TyG index (Fig. 1).

**Table 1:** Main characteristics of patients.

	TyG index < 8.65 group	TyG index ≥ 8.65 group	*P*
Age (yr)	56.5 ± 10.8	57.6 ± 11.1	NS
Men (n%)	77 (52%)	106 (52.5%)	NS
Weight (kg)	75.9 ± 5.9	75.8 ± 5.6	NS
HT (n%)	87(58.8 %)	127 (62.9%)	NS
HL (n%)	55 (37.2 %)	74 (36.6%)	NS
HDL mg/dL	44.9 ± 10.2	42.7 ± 9.9	.039*
TG mg/dL	188.5 ± 26.6	285.4 ± 72.4	<.001**
LDL mg/dL	129.4 ± 36.8	123.5 ± 42.0	NS
Fasting glucose mg/dL	115.2 ± 52.7	115.1 ± 53.0	NS
Smoker (n%)	42 (28.4%)	57(28.2%)	NS
Previous CVA (n%)	10 (6.8%)	15(7.4%)	NS
Previous Miyocardial Infarction(n%)	50(33.8%)	61 (30.2%)	.031*
Previous CABG(n%)	62 (23.9%)	30(28.8%)	NS
Creatinine before PCI (mg/dL)	1.00 ± 0.08	1.03 ± 0.09	NS
EF(n%)	53.7 ± 7.9	53.8 ± 7.5	NS
eGFR (mL/min 1,73²)	90.2 ± 16.0	88.3 ± 17.0	NS

CVA = cerebrovascular accident, GFR = glomerular filtration rate, EF = ejection fraction, HDL = high density lipoprotein, HL = hyperlipidemia, HT = hypertension, LDL = low density lipoprotein, NS = non significant, TG = triglyceride, TyG = triglyceride-glucose.

* Correlation is significant at the 0.05 level (2-tailed),

** Correlation is significant at the 0.001 level (2-tailed).

**Table 2:** Medication characteristics of patients.

Medication, n(%)	TyG index < 8.65 group	TyG index ≥ 8.65 group	*P*
Acetylsalicylic acid	73(49.3%)	96(47.5%)	NS
ACE I, ARB	77(52%)	111 (55%)	NS
Beta bloker	57 (38.5%)	76 (37.6%)	<.001
Statin	52 (35.1%)	70 (34.7%)	NS
Klopidogrel	12 (8.1%)	17 (8.4%)	NS
Furosemide	18 (6.9%)	11 (10.6%)	NS
Calcium channel blokers	17(11.5%)	21 (10.4%)	NS
Ticagrelor/Prasugrel	33 (22.3%)	44 (21.8%)	NS
Nitrates	26 (17.6%)	34 (16.8%)	NS

NS = non significant, TyG = triglyceride-glucose.

**Table 3:** In-hospital clinical course of patients.

	TyG index < 8.65 group	TyG index ≥ 8.65 group	*P*
Contrast volume mL	229.1 ± 17.4	228.4 ± 17.1	NS
Contrast nephropathy (n%)	14(9.5%)	42 (20.8%)	.004
Time to reperfusion h	4.5 ± 2.2	4.9 ± 2.2	NS
Troponin peak (ng/dL)	2.3 ± 0.9	2.2 ± 0.9	NS
Length of hospital stay (d)	4.6 ± 1.0	4.9 ± 1.8	NS
GRACE score	124.1 ± 10.7	123.3 ± 10.9	NS
Major bleeding (n%)	5 (3.4 %)	10(5%)	NS
Dialysis (n%)	3 (2%)	10 (5 %)	NS
Mechanical ventilation (n%)	4 (2.7%)	9(4.5%)	NS
In-hospital mortality(n%)	4 (2.7%)	12(5.9%)	NS
PCI (n%)	109 (73.6%)	155(76.7%)	NS
CABG (n%)	18 (11.5 %)	22 (10.9%)	NS
Medical treatment (n%)	21 (14.2%)	24 (11.9%)	NS

CABG = coronary aretry bypass grefting, NS = Non significant, PCI = percutaneus coronary intervention, TyG = triglyceride-glucose.

## 4. Discussion

CIN is a common complication after invasive treatment for NSTEMI, even in patients with normal baseline renal functions.^[22]^ This is associated with increased in-hospital mortality and longer hospital stays. Therefore, for high-risk patients, prompt risk assessment and timely prophylaxis are effective ways to reduce the incidence of CIN. Although the pathophysiological mechanisms of CIN are not fully understood, researchers suggest that CIN is caused by renal vasoconstriction, endothelial dysfunction, endothelial cell damage, subsequent renal tubular damage and medullary hypoxia.^[23,24]^ Older age, female sex, diabetes mellitus, congestive heart failure, and renal dysfunction are already known risk factors for CIN.^[25,26]^ Appropriate intravenous fluids, avoidance of nephrotoxic agents, selection of hypotonic or isotonic contrast media, and reduction of contrast media volume are all effective in reducing the incidence of CIN.^[27]^

This study investigated the predictive value of the TyG index for the development of CIN in non-diabetic NSTEMI patients after coronary angiography. We showed that TyG, a biomarker of insulin resistance, is an independent risk factor for CIN and has excellent predictive value in the diagnosis of her CIN in these patients.

Diabetes is an independent risk factor for CIN, and type 2 diabetes is primarily caused by impaired insulin secretion and insulin resistance.^[28]^ Insulin resistance is a systemic disease that affects multiple insulin regulatory pathways and many organs. It is characterized by decreased insulin action despite elevated blood insulin levels, increasing the risk of cardiovascular events.^[29]^ A recent study showed a role for insulin resistance in renal failure.^[30]^ Insulin resistance plays a role in increasing vascular permeability and is also associated with the development of early glomerular hyperfiltration, contributing to late glomerular injury in the early stages of diabetic nephropathy.^[31,32]^ Insulin signaling plays an important role in renal hemodynamics, podocyte viability, and tubular function. Therefore, hyperinsulinemia associated with insulin resistance is associated with inflammatory and adipocytokine disorders that can lead to glomerular damage.^[8]^ Hyperinsulinemia increases sodium reabsorption and glomerular filtration rate, ultimately leading to kidney damage.^[33]^ In addition, insulin resistance associated with oxidative stress ultimately leads to glomerulosclerosis and renal tubular interstitial damage.^[34]^ As indicators of insulin resistance, products of fasting triglycerides and fasting plasma glucose provide promising results that can be used to assess IR.^[12]^ The TyG index is a simple biomarker with high stability, and studies have shown that the TyG index outperforms HOMA in estimating IR.^[11,35]^ The TyG index has been shown to increase the risk of DM and various cardiovascular diseases.^[36–38]^ A recent study showed that; elevated TyG Index associated with CIN in NSTE-ACS patients implanted with DESs.^[39]^ In contrast to our study, this study includes only diabetic and stent implanted patients. Furthermore, our study includes all non-diabetic patients who underwent angiography and shows the correlation between CIN and TyG index in non-diabetic patients. The TyG index independently correlated with her CIN in patients without DM who underwent coronary angiography. The TyG index is expected to become a new predictor of CIN. Detection of insulin resistance in these patients using the TyG index and strict treatment will prevent the development of CIN and the occurrence of subsequent complications related to it.

Our study had some limitations. First, our study was a single-center study. Second, the study cohort was relatively small. Third, some risk factors for CIN, such as proteinuria and nephrotoxic agents, could not be fully evaluated. Finally, the hospitalized patient was not monitored and follow up for changes in her TyG index.

Our study shows that a high TyG index is closely correlated with a high incidence of CIN in non-diabetic NSTEMI patients undergoing coronary angiography. The TyG index is useful for risk stratification in clinical practice due to its simplicity and low cost. However, a mqulticenter study with a larger sample size is needed to further elucidate the correlation between the TyG index and her CIN incidence.

## Author contributions

**Conceptualization:** Erol Gürsoy.

**Data curation:** Onur Baydar.

**Formal analysis:** Erol Gürsoy, Onur Baydar.

**Investigation:** Erol Gürsoy, Onur Baydar.

**Methodology:** Erol Gürsoy.

**Resources:** Onur Baydar.

**Software:** Onur Baydar.

**Supervision:** Erol Gürsoy.

**Validation:** Onur Baydar.

**Visualization:** Erol Gürsoy.

**Writing – original draft:** Erol Gürsoy.

**Writing – review & editing:** Erol Gürsoy.
